# The Role of Citrate Transporter INDY in Metabolism and Stem Cell Homeostasis

**DOI:** 10.3390/metabo11100705

**Published:** 2021-10-15

**Authors:** Kavitha Kannan, Blanka Rogina

**Affiliations:** 1 Department of Genetics & Genome Sciences, School of Medicine, University of Connecticut Health Center, Farmington, CT 06030, USA; kkannan@uchc.edu; 2 Institute for Systems Genomics, School of Medicine, University of Connecticut Health Center, Farmington, CT 06030, USA

**Keywords:** *Indy*, SLC13A5, aging, intestinal stem cells, citrate transporter, longevity gene, metabolism

## Abstract

*I’m Not Dead Yet* (*Indy*) is a fly gene that encodes a homologue of mammalian SLC13A5 plasma membrane citrate transporter. Reducing expression of *Indy* gene in flies, and its homologues in worms, extends longevity. *Indy* reduction in flies, worms, mice and rats affects metabolism by regulating the levels of cytoplasmic citrate, inducing a state similar to calorie restriction. Changes include lower lipid levels, increased insulin sensitivity, increased mitochondrial biogenesis, and prevention of weight gain, among others. The INDY protein is predominantly expressed in fly metabolic tissues: the midgut, fat body and oenocytes. Changes in fly midgut metabolism associated with reduced *Indy* gene activity lead to preserved mitochondrial function and reduced production of reactive oxygen species. All these changes lead to preserved intestinal stem cell homeostasis, which has a key role in maintaining intestinal epithelium function and enhancing fly healthspan and lifespan. *Indy* gene expression levels change in response to caloric content of the diet, inflammation and aging, suggesting that INDY regulates metabolic adaptation to nutrition or energetic requirements by controlling citrate levels.

## 1. Introduction

*I’m not dead yet (Indy)* is a fly homologue of mammalian *SLC13A5* plasma membrane transporter with highest affinity for transporting the metabolite citrate, which is a critical regulator of metabolism [1,2,3]. *Indy* homologues have been identified in bacteria, worms, mice, rats, dogs, rabbits, monkeys, chimpanzees, zebrafish, pigs and humans [4,5,6]. The mammalian protein mINDY (also referred to as NaCT or Na^+^ coupled citrate transporter) is a plasma membrane citrate transporter similar to *Drosophila* INDY, but requires Na^+^ as a co-transporter [2,3,7,8]. The structural and functional characteristics of mouse and human mINDY are also distinct: the active sites that bind citrate and Na^+^ are different, and functionally, mouse mINDY is a low-capacity/high-affinity transporter, while human mINDY is a high-capacity/low-affinity transporter [9]. While differences exist between species, citrate transport is a conserved role for INDY. Citrate is a key metabolite in the tricarboxylic acid (TCA) cycle and has a highly conserved role in glucose, lipid, and energy metabolism across species. This is illustrated by studies that show similar effects of *Indy* reduction on metabolism in flies, worms, mice, rats, non-human primates and humans [10,11,12]. Reducing *Indy* gene expression levels extends longevity in flies and worms by affecting multiple metabolic pathways [1,10,13,14,15,16,17]. In this review, we focus on the effects of reduced INDY activity on metabolism, mitochondrial biogenesis and fly stem cell homeostasis that promote health and longevity. Table 1 includes abbreviations used in this review. 

## 2. *Indy* Reduction Extends Longevity of Flies and Worms by Mimicking Calorie Restriction

In flies, the INDY protein is predominantly expressed on the plasma membrane of metabolic tissues—the midgut, fat body, and oenocytes—all of which play an important role in uptake and storage of nutrients and energy production [1,2]. The worm homologues of INDY are predominantly expressed in the intestinal tract [15]. Mammalian mINDY is expressed at high levels in liver, brain, and testis, while low levels are found in heart, kidney, bones, and ovary in humans, mice, and rats [10,18,19,20]. The primary source of citrate comes from citrate synthesis in the mitochondria. However, in tissues where INDY is expressed, extracellular citrate is transported into cells through INDY, and regulation of this transport is important for maintenance of cytoplasmic citrate homeostasis [19,21].

Reduced expression of *Indy* in fruit flies or its homologue *CeNAC-2* in worms extends longevity [1,13,14,16,17,22]. Longevity in flies was extended in a number of independent *Indy* alleles, in which *Indy* mRNA levels are reduced by insertion of a P-element or a GFP protein trap in the first intron of the *Indy* gene [1,13,16,22,23]. Precise excision of the P-element in several *Indy* alleles resulted in a reversion to lifespan of control flies, confirming a causal relationship between P-element insertion in the *Indy* gene and fly longevity [1]. The presence of the P-element in the first intron of the *Indy* gene decreases the levels of *Indy* mRNA and protein levels, most likely by affecting transcription. In addition, natural populations of flies heterozygous for a *Hoppel* transposon insertion variant in the first intron of the *Indy* gene have reduced INDY levels and extended lifespan [24]. Three of these lines examined in detail were from Oahu, Hawaii (collected in 1955), Captain Cook, Hawaii (collected in 2007), and Hidalgo, Mexico (collected in 2005). Thus, heterozygosity for the *Hoppel* element has been preserved in laboratory conditions for many years conferring advantage of reduced *Indy* levels including increased fecundity [24]. Original studies using five independent *Indy* alleles, including *Indy^206^/+, Indy^302^/+,* and *Indy^159^*/+, showed longevity extension between 80 and 100% in both mean and maximal lifespan. Additional studies using *Indy^206^/+* showed that *Indy* reduction extends lifespan in different genetic backgrounds of flies including wild-type *Canton-S* stock, the *Hyperkinetic, Shaker, drop-dead* stocks, and short- and long-lived lines of Luckinbill [1,13,25]. Each of these stocks were separately maintained from the wild-type stocks for 45–50 years. Interestingly, *Indy* reduction further increases longevity of long-lived Luckinbill lines [13,26]. One of the original *Indy^206^* alleles was treated with tetracycline to avoid any influence of *Wolbachia* on lifespan and backcrossed into control *yw* and *w^1118^* genetic stocks for 10 generations. Compared to control stocks, longevity of *Indy^206^* allele was extended in *yw* background, but not in *w^1118^* background [13,22,27]. The influence of genetic background on longevity is well-known, as the effect of *Indy* reduction on longevity in *Drosophila* has been examined and documented in varied genetic backgrounds listed in the above mentioned studies [1,13,16,22]. Genetic background has been shown to impact quantitative physiological traits measured as markers of aging as well as intestinal stem cell proliferation in *Drosophila* [28,29]. Use of a diet similar to calorie restriction (CR) may affect longevity of *Indy* alleles in the Toivonen et al. study [22,27]. Studies using different *Indy* alleles (*Indy^159^, Indy^EP3044^, Indy^EP3366^, Indy^EY01442^, Indy^EY01458^, Indy^EY013297^*, *Indy^KG07717^*) backcrossed into the *yw* background showed that longevity extension is linked to the levels of *Indy* mRNA. A maximal increase in longevity of 34% was observed in heterozygous *Indy^206^/+, Indy^159^*/+ and *Indy^YC0030^*/+ alleles, in which *Indy* mRNA levels are about 55% lower compared to controls [13]. A small reduction of *Indy* mRNA leads to about 20% longevity extension, while a non-significant reduction of *Indy* mRNA levels does not significantly affect *Indy^EP3366^*/+ lifespan compared to controls. 

*Indy* reduction regulates cytoplasmic citrate levels, which affects cellular metabolism and energy production in mitochondria. Reducing *Indy* expression lowers available energy resources and creates a state similar to CR. Accordingly, flies with reduced *Indy* levels have several metabolic and physiological phenotypes parallel to CR [30,31]. These changes include increased mitochondrial biogenesis, increased spontaneous physical activity, increased resistance to stress, reduced reactive oxygen species (ROS) production, smaller body weight and reduced insulin/insulin-like growth factor signaling (IIS), among others [16,22,23,32]. IIS is an evolutionarily conserved pathway involved in growth and metabolism. Reduced IIS extends lifespan in multiple species [33,34,35]. In *Drosophila*, the IIS pathway is activated under physiological conditions when nutrients are abundant and *Drosophila* insulin-like peptides (DILPs 1–8) bind to the insulin receptor (InR), which activates the downstream members of the IIS pathway. Activation of IIS results in phosphorylation of a transcription factor from the Forkhead box-O family (FoxO), which prevents its nuclear localization [36,37]. Low nutrient levels downregulate IIS, which leads to nuclear localization of unphosphorylated FoxO where it activates transcription of antioxidant genes and *dPGC-1/spargel*, a *Drosophila* homologue of peroxisome proliferator-activated receptor gamma co-activator-1 (PGC-1). IIS is reduced under CR conditions and in *Indy^206^/+* flies [16,22]. This is illustrated by findings that control flies on a CR diet and *Indy^206^/+* flies on a high calorie diet have FoxO predominantly localized in the nuclei of fly fat bodies, which is indicative of reduced IIS [22,38]. In addition, the levels of *dilp2, dilp3*, and *dilp5* were reduced in the heads and thoraces of *Indy^206^/+* flies to identical levels found in CR flies [22]. Similarly, increased insulin sensitivity was found in *mINDY^−^/^−^* knockout (*mINDY-KO*) mice, suggesting conserved INDY effects on insulin signaling and metabolic regulation [18].

Moreover, the longevity of *Indy^206^/+* heterozygous flies is not further extended by CR, which is consistent with *Indy^206^/+* flies already being in a state of CR when kept on a regular diet [22,23]. While *Indy^206^/Indy^206^* homozygous flies live longer than controls on a regular and a high calorie diet, they live shorter on a CR diet, most likely due to starvation. Consistent with physiological changes in CR, studies in worms with reduced *Indy* homologue, *CeNAC-2* show reduced body fat content and body size [14,17]. Likewise, *mINDY-KO* mice have decreased body weight, increased hepatic mitochondrial biogenesis, increased mitochondrial density, increased insulin sensitivity, higher lipid oxidation, and similar hepatic transcriptional changes to those found in liver of CR mice [10,18]. In rats, a reduction of *mIndy* levels with antisense oligonucleotides protects animals from harmful effects of diet-induced hepatic steatosis and insulin resistance [39,40]. Identification of similar phenotypes and changes associated with reduced INDY levels in multiple organisms suggests a highly conserved role of INDY in metabolism.

## 3. The Role of INDY in Metabolism

**Figure 1:**
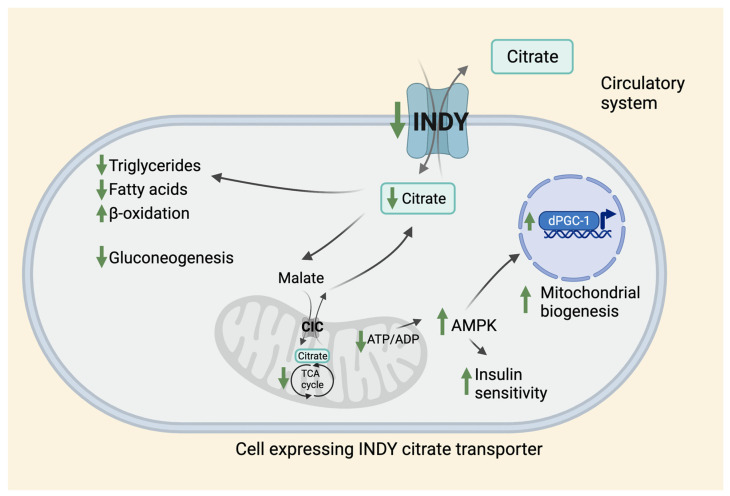
Effects of citrate transporter on metabolism. INDY is a plasma membrane citrate transporter that transports citrate between the circulatory system and the cytosol of the INDY-expressing cell in *Drosophila* and other species. Reduced INDY activity results in lower cellular levels of citrate that affects metabolism leading to reduced synthesis of triglycerides, and fatty acids, increased β-oxidation, and decreased gluconeogenesis. Citrate is converted to malate in the cytosol and is transported to mitochondria through CIC located on the inner mitochondrial membrane, where it is used in the TCA cycle. The citrate synthesized in the mitochondria is then transported back to the cytosol through CIC in exchange for malate. Low INDY levels lead to a decrease in ATP/ADP ratio that activates AMPK, which promotes insulin sensitivity and mitochondrial biogenesis via upregulation of dPGC-1 levels. CIC: Citrate/isocitrate carrier.

In flies, INDY is a plasma membrane transporter for TCA cycle intermediates [2]. Functional studies in frog oocytes show that INDY has a role as an anion exchanger, and exchanges citrate-citrate, citrate-succinate, citrate-oxaloacetate, but also succinate-α-ketoglutarate, and succinate-fumarate [2]. Taken together, fly INDY is an exchanger of dicarboxylate and tricarboxylate TCA-cycle intermediates. Exchange of internal anion for external citrate is pH-dependent but Na-independent. Thus, INDY transports citrate bidirectionally between the midgut and hemolymph (hemolymph is analogous to blood that acts as a circulatory fluid in flies). Citrate in hemolymph circulates to other metabolic tissues, mainly the fat body and oenocytes. In mammals, mINDY transports citrate from circulation across the plasma membrane, dominantly in the liver, brain, and testis [11,41]. In liver, mINDY is present in the sinusoidal membrane facing the blood [11,41]. INDY levels regulate glucose and lipid metabolism and energy production by affecting cytoplasmic citrate levels (Figure 1). 

Cytosolic citrate is broken down to acetyl-CoA and oxaloacetate by the enzyme ATP citrate lyase (ACLY). Cytoplasmic acetyl-CoA can be used as a building block for fatty acids, triacylglycerols, cholesterol, and low-density lipoprotein synthesis [42,43,44]. When citrate is metabolized to malate, it enters mitochondria where it is used in the TCA cycle for generation of NADH and FADH_2_, which can enter the mitochondrial matrix for energy production. When ATP production is high, mitochondrial citrate is transported by mitochondrial citrate transporter CIC (Citrate/isocitrate carrier; also known as SLC25A1) into the cytosol in exchange for malate from cytoplasm into the matrix [45,46]. Under physiological conditions when required ATP levels are met, citrate can be used in catabolism. Cytoplasmic citrate levels affect glucose levels by inhibiting the activity of rate-limiting enzyme phosphofructokinase-1 and slowing down glycolysis, while also activating fructose 1,6 bisphospatase to stimulate gluconeogenesis. Cytosolic citrate also downregulates fatty-acid β-oxidation pathways and is an allosteric activator of acetyl-CoA carboxylase beta isoform (ACCbeta), which affects transfer of fatty acids to mitochondria and its use in energy production. Reduced INDY transporting activity leads to low acetyl-CoA levels available for fatty acid and cholesterol synthesis, and also releases the inhibitory effects of citrate on glycolysis, thereby decreasing gluconeogenesis. Reduced INDY levels lead to reduced ATP production, and increased ADP levels that activate AMPK, a cell nutrient sensor. AMPK promotes insulin sensitivity, lipid-oxidation, and mitochondrial biogenesis by activating mitochondrial transcriptional co-activator *dPGC-1/spargel* [18,47]. AMPK also phosphorylates ACCbeta and inhibits its activation by citrate and its basal activity. Similar physiological changes are associated with adaptation to low nutrients during CR in a wide variety of species. Effects of *Indy* reduction on metabolism have been observed in flies, worms, mice, rats, non-human primates, and humans [10]. As noted in the previous section, reducing CeNAC-2, worm homologues of INDY lowers lipid levels, reduces weight, and activates AMPK [17]. *mINDY-KO* mice have reduced hepatic lipogenesis, enhanced hepatic lipid oxidation, increased energy expenditure, increased mitochondrial biogenesis, increased insulin sensitivity, and are prevented from weight gain and protected from high-fat diet-induced steatosis and insulin resistance [18]. Adult rats aged on a high-fat diet have lower hepatic lipid levels and increased insulin sensitivity when liver-specific mINDY activity was reduced by using antisense oligonucleotides [39]. 

The role of mINDY in metabolism is corroborated by severe phenotypes caused by mutations in mammalian *mINDY*. The *mIndy* gene encodes the only known neuronal plasma membrane citrate transporter expressed in the hippocampus, cerebellum, cerebral cortex, and olfactory bulb [20,48]. A loss of function of *mIndy* (*mSLC13A5*) and mutations in the coding region of the *mIndy* gene in humans is the cause of an autosomal recessive disorder referred to as infantile epileptic encephalopathy. This disorder causes neonatal epilepsy with seizures during the first few days of a newborn’s life as well as developmental delays, limited speech, tooth defects, and limited motor skills [49,50]. There are about 41 different mutations in the *mIndy* gene, which are all associated with infantile epileptic encephalopathy [9,11]. Kohlschütter-Toönz syndrome (KTS) is another disorder caused by *mIndy* (*mSLC13A5*) mutations, which has similar phenotypes to infantile epileptic encephalopathy and the mechanism underlying KTS is unknown [51]. Mutations in *mIndy* are classified based on their effects on mINDY protein function as transporter or on its stability, but all *mIndy* mutations result in loss of citrate transport [50]. Lack of citrate transport affects neuronal energy production, neurotransmitter biosynthesis, as well as formation of the myelin sheath in neurons [11,19]. The original study on *mINDY-KO* mice did not find any abnormal phenotype that could suggest a seizure phenotype [18]. However, detailed examination of *mINDY-KO* mice using video-electroencephalography (EEG) monitoring showed that a fraction of *mINDY-KO* mice had a propensity for epileptic seizures and proepileptic excitability changes [52]. In contrast, another recent report that used a different *mINDY-KO* mouse model described increased motor coordination and improved recognition and social memory, which were similar to CR mice [53]. The role of mINDY in metabolism is further underscored by studies that show that silencing mINDY in human hepatocarcinoma cells decreases their proliferation [54,55,56].

## 4. *Indy* Transcriptional Regulation

The importance of citrate in metabolic regulation is underscored by reports that *Indy* transcription is regulated by nutritional status of the organism. *Indy* gene expression in the midgut is regulated by diet and stress. *Indy* mRNA levels are increased in aged control flies and in flies exposed to a high nutrient diet and oxidative stress. In contrast, control flies raised on a CR diet, and *Indy^206^/+* heterozygous and *Indy^206^/Indy^206^* homozygous flies on a standard diet have reduced *Indy* mRNA levels [16,22]. In primary rat hepatocytes, *mIndy* transcription is induced during starvation by glucagon hormone via binding of the transcription factor cAMP-responsive element-binding protein (CREB) to its binding sequence in the *Indy* promoter [57]. This induction results in increased INDY transporting activity that leads to increased cytosolic citrate levels and its utilization in gluconeogenesis and lipid synthesis. Studies in human and rat primary liver cells have shown that *mIndy* transcription is upregulated by binding of rifampicin and the benzo[a]pyrene to the nuclear receptor pregnane X receptor (PXR) and aryl hydrocarbon receptor (Ahr), respectively [58,59]. Consistent with the metabolic role of INDY, increased *mIndy* expression levels lead to hepatic lipid accumulation. Furthermore, increased levels of *mIndy* expression are associated with non-alcoholic fatty liver disease (NAFLD) in obese, insulin-resistant humans [60]. This increase is caused by binding of the inflammatory interleukin-6 (IL-6), released from adipocytes, to the IL-6 receptor, which leads to phosphorylation and nuclear transfer of the transcriptional factor STAT3 and its binding to the STAT3 binding sequence in the *mIndy* promoter [60]. Moreover, inhibition of liver *mIndy* (*mSLC13A5*) by RNA interference (RNAi) prevents diet-induced NAFLD and improves insulin sensitivity in mice [40]. *mIndy* expression is upregulated in the liver cells of nonhuman primates fed for 2 years with a high-fat and high-sucrose diet. Their liver cells also had high levels of circulating lipids and glucose [60]. Taken together, INDY levels have profound effects on cellular metabolism that have been implicated in human NAFLD and obesity models in non-human primates and mice.

## 5. Effects of Reduced *Indy* on Intestinal Stem Cell Homeostasis

INDY is predominantly expressed in the basolateral membrane of the fly midgut, a tissue actively involved in metabolism. Therefore, it is essential to determine how INDY reduction affects intestinal stem cell (ISC) physiology and if these changes contribute to extended longevity. In flies and mammals, the gastrointestinal (GI) tract is involved in many physiological processes including nutrient digestion, nutrient absorption, protection against microbiota, endocrine function, and excretion [61]. The mammalian GI tract consists of ISCs that are self-renewing and give rise to progenitor cells that differentiate into several types of cells in the intestinal epithelium. Differentiating ISCs replace damaged cells to maintain gut integrity and function, which is vital for healthy aging [62,63]. The *Drosophila* gut has been an excellent system to study cellular mechanisms and biological processes involved in gut maintenance and homeostasis, due to similar physiological processes that occur in the intestines of flies and mammals [64]. The adult *Drosophila* gut has three regions: the foregut, midgut, and hindgut. The *Drosophila* midgut is analogous to the small intestine and contains multipotent ISCs, progenitor enteroblasts (EBs), enterocytes (ECs), and entero-endocrine cells (EEs) along with the visceral muscle (VM), which lines the intestinal epithelium along with the basement membrane. The EBs are post-mitotic cells that arise from the ISCs and differentiate into ECs, which have absorptive function and EEs, which have a role in secretion of gut hormones [65,66,67]. 

**Figure 2:**
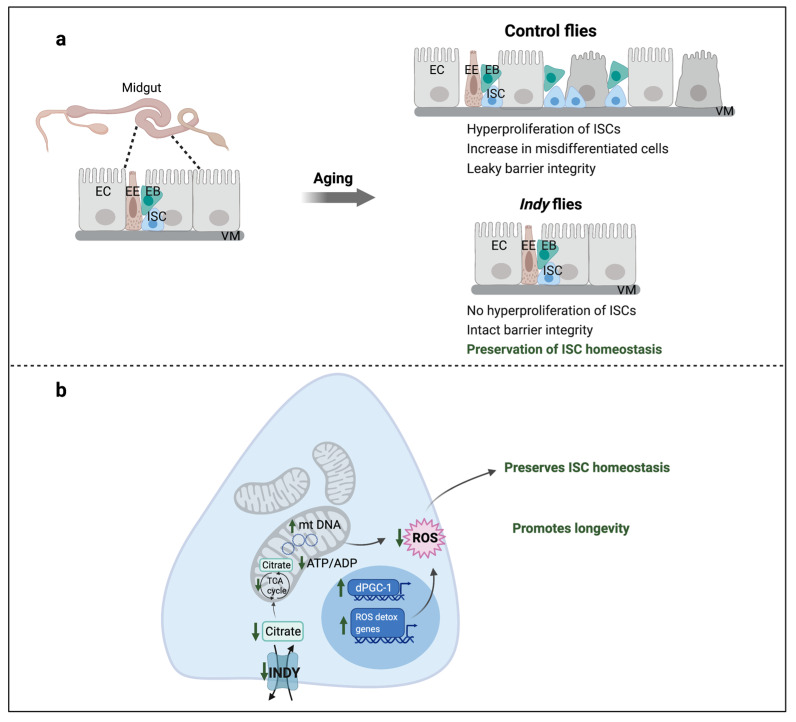
*Indy* reduction preserves ISC homeostasis. (**a**) Aging causes disruption of intestinal epithelium that results in loss of barrier integrity and uncontrolled proliferation of ISCs and accumulation of mis-differentiated ISCs resulting in loss of gut homeostasis; (**b**) *Indy* reduction in the midgut results in reduced cytosolic citrate levels, which increases mitochondrial biogenesis through upregulation of dPGC-1, increases mitochondrial DNA and number, and upregulates ROS-detoxification genes that reduces overall cellular ROS levels. This preserves ISC homeostasis and midgut integrity leading to a delay in intestinal aging thereby promoting longevity. ISC: Intestinal stem cell; EB: Enteroblast; EC: Enterocyte; EE: Enteroendocrine cell; VM: Visceral muscle; ROS: Reactive oxygen species.

Aging is characterized by a gradual decrease in mitochondrial biogenesis caused by reduced expression of *dPGC-1/spargel* mRNA. dPGC-1 promotes mitochondrial biogenesis by activating expression of genes encoding mitochondrial proteins [16,68,69]. *Indy**^206^***/+ and *Indy**^206^***/*Indy**^206^*** flies have increased *dPGC-1* mRNA levels in heads, thoraces, and midguts that lead to increased gene expression of electron transport chain (ETC) components and increased mitochondrial copy number (Figure 2) [16]. *Indy**^206^***/+ and *Indy**^206^***/*Indy**^206^*** flies have lower levels of mitochondrial ROS production and increased expression levels of ROS-detoxification genes, *Glutathione S transferase E1 (GstE1)* and *Glutathione S transferase D5 (GstD5),* which contribute to their increased resistance to oxidative stress. Moreover, mitochondria isolated from the midguts of *Indy**^206^***/+ and *Indy**^206^***/*Indy**^206^*** flies have high mitochondrial membrane potential that confirm preservation of mitochondrial function. Interventions that increase ETC function and regulate mitochondrial biogenesis through overexpression of *dPGC-1* in the gut have led to preserved ISC homeostasis in older flies and extended lifespan in *Drosophila* [16,68,69,70]. Genetic epistasis studies showed that longevity was not extended in *Indy^206^; dPGC-1^KG08646^* flies with both reduced *Indy* and *dPGC-1* levels. In addition, overexpression of *dPGC-1* in *Indy**^206^***/+ flies did not further extend the long life of *Indy**^206^***/+ flies, indicating that *Indy* and *dPGC-1* longevity pathways overlap [16].

An age-associated increase in ROS levels in the midguts of flies activates ISC proliferation rate leading to uncontrolled hyperproliferation of ISCs and accumulation of mis-differentiated and damaged cells [61]. *Indy**^206^***/+ and *Indy**^206^***/*Indy**^206^*** flies have lower ROS levels compared to control flies, resulting in reduced ISC proliferation rate relative to controls at all ages, which was determined by the number of escargot-positive cells, a marker for ISCs and progenitor EBs (Figure 2). Actively proliferating cells in the midgut, measured as phosphorylated histone H3 (pH3)-positive cells, are also reduced in *Indy* flies indicating a delay in age-associated increase in ISC proliferation rate. These findings illustrate that *Indy* flies have preserved stem cell differentiation and ISC homeostasis [16]. It has been shown that CR prevents age-associated ISC hyperproliferation that contributes to extended lifespan [71]. It is possible that reduced *Indy* mRNA in CR flies contributes to preserved ISC homeostasis. 

These studies suggest a link between ISC proliferation rate and *Indy* mRNA expression levels in response to nutrients, stress, and aging. It is highly likely that during aging and stress, ISCs have increased energy demands required for proliferation to replace aberrant cells, and a rise in INDY levels would increase cytoplasmic citrate levels that would be utilized for energy production in mitochondria. The role of mINDY in cell proliferation is supported by findings that RNAi mediated silencing of *mINDY (mSLC13A5*) in human hepatoma cell lines (HepG2) dramatically suppressed cell proliferation and colony formation [54,56]. The growth of HepG2 cell-derived xenographs containing mINDY-shRNA in nude mice was also decreased. Inhibitory effects of reduced *mIndy* contributed to decreased lipogenesis and decreased long-chain fatty acid and cholesterol synthesis, which are required for growth. Hepatocarcinoma proliferation suppression by *mIndy* inhibition may also be mediated by inhibitory effects of activated AMPK on the oncogenic mTOR signaling pathway [72]. 

With aging, there is a decline in the integrity of intestinal epithelium that acts as a barrier between intestinal lumen and hemolymph in the gut, and factors that maintain its structure and function are critical to promote longevity. Aged *Indy**^206^***/+ and *Indy**^206^***/*Indy**^206^*** flies exhibit intact intestinal structure and integrity revealed by electron micrographs. When these flies were fed with food containing non-absorptive blue dye, blue dye was only observed in the proboscis and gut [16]. In contrast, blue dye was observed in all tissues of control flies; because of a loss of intestinal integrity with aging, the dye can easily move from lumen of the gut to hemolymph and the rest of the body, resulting in what has been referred to as a “smurf” phenotype. Overall, this indicates that reducing INDY activity in the midgut preserves ISC homeostasis, enhances intestinal health, and promotes longevity. Molecular mechanisms that couple metabolism and ISC proliferation play a significant role in longevity, emphasizing the importance of future research to fully understand INDY’s role as a link between these key processes. It is most likely that beneficial effects of reduced *Indy* on ISC homeostasis and metabolism in other tissues have a synergistic effect on fly health and longevity. While effects of *Indy* as well as *mIndy* reduction on metabolism in cells expressing INDY on their plasma membrane are highly conserved across species, mINDY is not present in the human GI tract. Therefore, it is highly likely that other SLC family transporters play the role of fly *Indy* in the human GI tract.

## 6. Effects of *Indy* Reduction on Spermatogenesis

A recent report by Hudry et al. highlights the role of INDY and citrate in interorgan communication and sperm maturation [73]. Interestingly, this communication is mediated by close spatial organization of fly testis and midgut. In *Drosophila*, the midgut consists of five distinct regions from anterior to posterior direction marked as R1–R5 [74,75]. JAK/STAT signaling is activated in the R4 midgut region by the cytokine Upd1 released from the testis soma and this has been shown to increase intestinal sugar gene expression to produce cytosolic citrate. INDY transports citrate from the R4 region to the testis and promotes maturation of sperm. Moreover, impaired citrate efflux in *Indy* knockdown flies negatively affects sperm maturation and decreases spermatocyte numbers by causing metabolic changes in testis. In males, citrate serves as a signal that regulates sex specific differences in sugar gene expression but also increases food uptake. Taken together, this study presents evidence that by affecting citrate levels INDY plays a role in interorgan communication between the gut and testis in a sex-dependent manner [73]. Therefore, it would be worth examining the role of mINDY in testis of mice, rats and humans, where INDY is expressed at low levels [10,19]. Effects of obesity, high-fat diet, and glucose and lipid metabolism, and the role of mitochondria on spermatogenesis in human males have been described [76,77]. 

The ability of *Indy* reduction to decrease spermatogenesis might seem contradictory to increased fecundity observed in *Indy^206^/+* and *Indy^302^/+* females [1,23,73]. However, we have previously examined a possibility that *Indy* reduction might affect male and female physiology differently, which could influence fecundity and longevity [1,23]. We determined fecundity of at least 23 mating pairs, each pair with a single male and female heterozygous for *Indy^206^/+* and *Indy^302^/+* and compared them to control flies (*CS-5*). Heterozygous F1 *Indy^206^/+* and *Indy^302^/+* flies were generated by two different crosses: the first where parental males were *Indy^206^/Indy^206^* or *Indy^302^ /Indy^302^* homozygous and females were *CS-5* flies, and in the second where parental females were *Indy* homozygous flies (same lines) and males were *CS-5* flies [1,23]. Heterozygous *Indy^206^/+* and *Indy^302^/+* flies, generated by both crossing schemes, laid more eggs compared to control flies with no significant difference between crosses [1]. No differences in metabolic rate or flight velocity were observed in INDY heterozygous flies from either crossing schemes [23]. Beneficial effects on female fecundity were also observed in three independently natural populations of flies containing the *Hoppel* element in the *Indy* gene region [24]. As noted in Section 2, while *Indy^206^/+* and *Indy^302^/+* fecundity was higher on a standard and high calorie diet, it was lower compared to controls when flies were kept on a CR diet, most likely due to low energy available for egg production, which is consistent with *Indy* reduction creating a state similar to CR [23]. 

It is worth noting that fly mating is a complex process that affects behavior, physiology, and longevity of female flies [78,79,80]. During mating, a male fly transfers hundreds of sperm to a female fly, who stores sperm in spermathecae and seminal receptacle for up to 2 weeks. Females are able to release sperm from storage and fertilize their own eggs for 2 weeks after a single mating. During mating, about 100 seminal fluid proteins (SFPs) are transferred together with sperm, which affect sperm storage, egg production, release of the sperm for fertilization, and other aspects of female physiology [78]. The numbers of spermatocytes were not determined in *Indy^206^/+* and *Indy^302^/+* flies; however, even if the number of sperm transferred from a male to female fly was lower compared to controls, the female flies would still likely have more than enough sperm to fertilize their eggs. Once the sperm and SFPs that prevent mating are depleted, female flies could mate again. In summary, while effects of *Indy* reduction on spermatogenesis and female egg production illustrate sex-specific effects, no other sex-specific differences were found in examined physiology, mortality rate or longevity but future detailed analyses in this area could reveal new insights.

## 7. Conclusions and Future Directions

Reduced gene expression of fly *Indy* and its worm homologues extends their life span by altering metabolism in a manner similar to CR. Fly INDY and homologues in worms and mammals share a preference for transporting citrate. By regulating cytoplasmic citrate levels, INDY acts as a metabolic regulator in modulating glucose and lipid levels, and energy production in mitochondria [44,81]. Metabolic changes associated with *Indy* reduction in the fly midgut results in dramatic changes in midgut physiology that lead to preserved intestinal ISC homeostasis. This is vital for replacement of damaged cells and the maintenance of midgut function illustrated by preserved intestinal integrity [16,44]. *Indy* reduction extends lifespan in male and female flies, but the effects of *Indy* reduction on ISC homeostasis have only been studied in female flies. Male and female flies have different gut pathologies and respond differently to stress and CR, with males having a delay in age-related gut pathology and lower ISC proliferation, while females respond better to stress and CR [82,83]. Considering these differences, it would be of interest to determine the effects of *Indy* reduction on the midguts of male flies. ISC homeostasis is regulated by multiple signaling pathways including IIS, Notch, EGF, Wnt/wingless, BMP/Dpp, JNK, and JAK/STAT, among others [62,84,85,86]. It would be important to assess the status of different signaling pathways in flies with reduced *Indy* expression, as metabolic changes might delay age-associated activation of these pathways and could contribute to preservation of ISC homeostasis and longevity. The data reviewed here support the role of INDY as a metabolic regulator: *Indy* expression changes in response to nutrient availability and requirements of the organism, which, by regulating citrate levels, controls energetic status of the organism to maintain tissue-specific metabolic requirements leading to preserved organismal health and homeostasis. Reduced INDY levels in the midgut could then prevent age-related ISC hyperproliferation by reducing the available energy for proliferation.

**INDY as a potential therapeutic target**

Aging can be modulated by genetic and environmental manipulations such as CR [87]. Reduced gene expression of fly *Indy* and its worm homologues extends their life span by altering metabolism in a manner similar to CR. *Indy* reduction decreases the rate of aging, as measured by a decrease in the slope of the mortality curve [23]. Thereby, pharmaceutical interventions that reduce INDY could mimic the benefits of CR without reducing caloric uptake. Studies in flies, worms, mice, and rats have made INDY a potential target that could be used in a clinical setting to alleviate some metabolic disorders such as NAFLD, Type 2 diabetes, insulin resistance, and even carcinogenesis. In brief, liver-specific *mIndy* RNAi silencing prevents diet-induced NAFLD in mice [40]; *mINDY-KO* mice are protected from age- and diet-induced insulin insensitivity and obesity; while antisense oligonucleotides given to rats on a high-fat diet prevent hepatic steatosis and hepatic insulin resistance [39]. Particularly promising is use of mINDY inhibitors in hepatocarcinoma cells due to their effects on energy required for proliferation and negative effects on oncogenic mTOR signaling. Recent studies in *mINDY-KO* mice indicate potential use of mINDY inhibitors for regulating blood pressure, increasing motor coordination, and promoting social and recognition memory in mice [12,53,88]. There is also an urgent need to study the mechanisms of negative effects of *mIndy* mutations in neonatal epilepsy in model organisms expressing *mIndy* human mutations to provide insights for future therapies. Altogether, the physiological function of INDY and its effects on metabolism are highly conserved across organisms, making INDY a compelling therapeutic target for preservation of metabolic homeostasis. 

## Figures and Tables

**Table 1:** List of abbreviations.

Abbreviation	Description
ACLY	ATP citrate lyase
AhR	Aryl hydrocarbon receptor
AMPK	5′AMP-activated protein kinase
CeNac2	*Caenorhabditis elegans* sodium cotransporter 2
CIC	Citrate/isocitrate carrier also known as SLC25A1
CR	Calorie restriction
CREB	cAMP responsive element-binding protein
DILPs	*Drosophila* Insulin-like peptides
EB	Enteroblast
EC	Enterocyte
EE	Entero-endocrine cell
ETC	Electron transport chain
FoxO	Transcription factor from the Forkhead box-O family
*GstD5*	*Glutathione S transferase D5*
*GstE1*	*Glutathione S transferase E1*
HepG2	Hepatoma G2 cell line
*Hoppel*	Transposable insertion variant
IIS	Insulin/insulin-like growth factor signaling pathway
IL-6	Interleukin 6
*Indy*	*Drosophila* I’m not dead yet gene
ISC	Intestinal stem cell
*mINDY*	Mammalian homolog of *Indy* (also referred as mSLC13A5/NaCT)
*mSLC13A5*	Mammalian Sodium-dicarboxylate cotransporter solute carrier family 13, member 5 (also referred as *mINDY*/NaCT)
NaCT	Sodium (Na+) coupled citrate transporter (also referred as *mSLC13A5; mINDY*)
NAFLD	Non-alcoholic fatty liver disease
*dPGC-1/spargel*	*Drosophila* homologue of peroxisome proliferator-activated receptor gamma co-activator-1 (PGC-1)
PXR	Pregnane X receptor
ROS	Reactive oxygen species
*SLC13A5*	Sodium-dicarboxylate cotransporter solute carrier family 13, member 5 (also referred as INDY/NaCT)
STAT3	Signal transducer and activator of transcription 3
TCA	Tricarboxylic acid cycle
VM	Visceral muscle
